# Genetic Screening—Emerging Issues

**DOI:** 10.3390/genes15050581

**Published:** 2024-05-03

**Authors:** Martina C. Cornel, Karuna R. M. van der Meij, Carla G. van El, Tessel Rigter, Lidewij Henneman

**Affiliations:** 1 Section Community Genetics, Department of Human Genetics, Amsterdam UMC, Vrije Universiteit Amsterdam, 1007 MB Amsterdam, The Netherlands; 2 Amsterdam Public Health Research Institute, 1100 DD Amsterdam, The Netherlands; 3 Amsterdam Reproduction and Development Research Institute, 1100 DD Amsterdam, The Netherlands

**Keywords:** genetic screening, neonatal screening, prenatal screening, genetic carrier screening, cascade screening, screening criteria, DNA sequencing

## Abstract

In many countries, some form of genetic screening is offered to all or part of the population, either in the form of well-organized screening programs or in a less formalized way. Screening can be offered at different phases of life, such as preconception, prenatal, neonatal and later in life. Screening should only be offered if the advantages outweigh the disadvantages. Technical innovations in testing and treatment are driving changes in the field of prenatal and neonatal screening, where many jurisdictions have organized population-based screening programs. As a result, a greater number and wider range of conditions are being added to the programs, which can benefit couples’ reproductive autonomy (preconception and prenatal screening) and improve early diagnosis to prevent irreversible health damage in children (neonatal screening) and in adults (cancer and cascade screening). While many developments in screening are technology-driven, citizens may also express a demand for innovation in screening, as was the case with non-invasive prenatal testing. Relatively new emerging issues for genetic screening, especially if testing is performed using DNA sequencing, relate to organization, data storage and interpretation, benefit–harm ratio and distributive justice, information provision and follow-up, all connected to acceptability in current healthcare systems.

## 1. Introduction

Genetic screening may be defined as any kind of medical test performed for the systematic early detection or exclusion of a hereditary disease, the predisposition to such a disease or to determine whether a person carries a predisposition that may produce a hereditary disease in offspring [1]. For decades, genetic screening has been offered in many countries, either in population screening programs offered to all or part of the population at higher risk for disease, or in a less formalized way. Screening can have benefits, but can also cause harm, including false positive screening results, overtreatment, psychological harm or physical harm from the screening test itself or from confirmatory diagnostic follow-up examinations.

It has been said that genetic screening should “proceed with caution” [1]. While most of its ethical issues may be similar to those associated with other screening tests, including challenges with determining cost-effectiveness and organizing the responsible implementation of testing healthy individuals in a population [2], genetic screening concerns traditionally relate specifically to the possible consequences of a genetic test result for other family members, the possibility of genetic discrimination or stigmatization based on the results and the fact that screening does not necessarily lead to disease prevention or treatment [1]. 

Countries and jurisdictions have different decision-making procedures for determining whether the benefits outweigh harms in population-based screening programs, and thus whether these programs should be implemented and funded or not. Although decision-making policies may vary between countries, these procedures often build on the principles proposed by Wilson and Jungner for the World Health Organization in 1968 [3]. In the age of genomics, these screening criteria were revisited [4]. While the original publication in 1968 focused on early diagnosis and the availability of treatment, the revisited criteria also touch upon prenatal screening and the availability of reproductive options, making informed choices and respect for autonomy an even more important issue [4,5]. These currently widely used criteria describe, e.g., that there should be evidence of screening program effectiveness, and that the program should promote equity and access to screening for the entire target population [1]. 

Technological advancement in the development of new genetic tests and new treatment options are instigating rapid change, i.e., constant dynamics, and ever-increasing pressure, in the field of genetic screening. In a previous paper, we described how these dynamics influence the fields of neonatal versus carrier screening [5]. We discussed converging debates about potential beneficiaries (newborn, family, society) and the goals and consequences of screening (disease prevention versus reproductive autonomy). We called for “an exchange of vision and knowledge among all stakeholders [in the fields of newborn and carrier screening] to attune the dynamics of screening” [5]. 

Having a closer look at the stakeholders involved and their perspectives may be even more relevant today, since new techniques are entering the field of screening. For instance, whole genome sequencing is being introduced in pilot studies for neonatal screening, potentially replacing some laboratory testing of metabolites [6], and facial recognition may be implemented to screen for genetic syndromes [7]. New techniques challenge stakeholders to constantly attune to each other, especially where there are limited budgets, uncertainties and a lack of experience with the responsible application of new technologies. Meanwhile, some potentially useful existing applications of genetic screening still encounter practical challenges to their optimal implementation; these include newborn screening [8] and cascade screening in blood relatives for a variant that confers an inherited disease [9,10]. 

**Figure 1:**
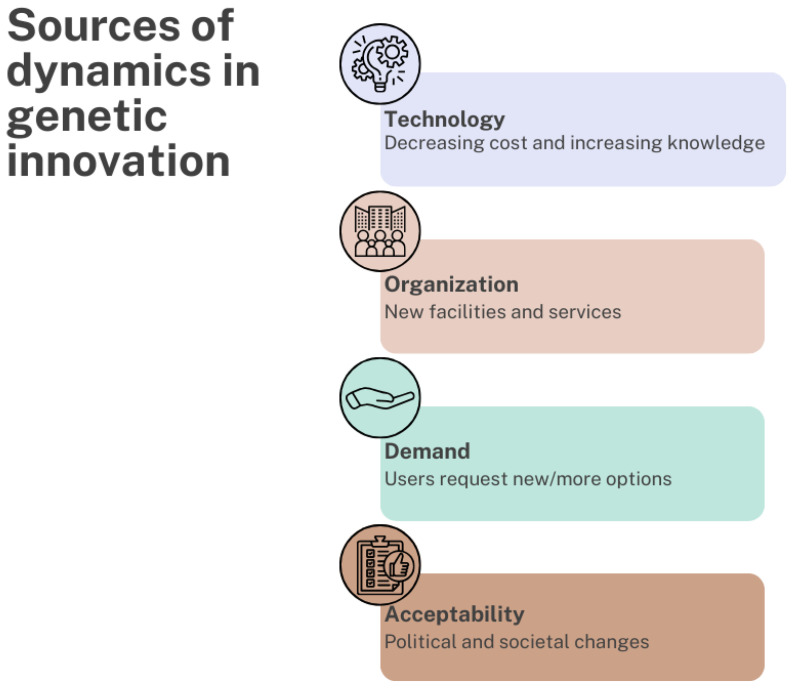
Sources of dynamics in genetic screening innovations (adapted from [11]).

Major drivers of innovation in genetic screening are the decreasing costs of genome sequencing and the increasing understanding of genetic variants, allowing for genetic technologies to be increasingly used in screening programs. Consequently, changes in how test offers are organized may be needed to develop facilities and services (e.g., offering genetic tests from general screening laboratories instead of clinical genetic laboratories). Users may or may not demand or accept the new options, and their implementation is influenced by political and cultural acceptability. Together, these sources of dynamics in genetics can drive change (Figure 1).

The extent to which these changes are disruptive to the existing (public) healthcare system greatly influences the speed of implementation of the innovation. Some new technological options are being implemented rather fast, mainly due to facilitating factors. This was, e.g., the case with the introduction of prenatal screening for aneuploidies using non-invasive prenatal testing (NIPT), based on the analyses of cell-free DNA in maternal blood. The implementation of this screening has benefitted from user demand, the organizational structure of previously established prenatal screening programs and the start of “local niches” to become acquainted with the technology [12]. Other technological options for genetic screening have encountered more hurdles relating to financial, cultural and religious factors, or to the need to establish completely new practices such as preconception carrier screening for recessive disorders [13]. In screening, there are always disadvantages. The discussion on the balance of pros and cons of each new technological option may demand even more attention when DNA testing is being considered, and more results could be generated than current targeted tests provide [14]. In this paper, we discuss recent emerging issues in the field of genetic screening. We will discuss (1) key developments in genetic screening, and (2) challenges faced by screening tests offered at different phases in life.

## 2. Key Developments in Genetic Screening

### 2.1. More Treatments, More Genetic Variants to Test

In our previous paper, we discussed treatment-driven and technology-driven developments in neonatal and preconception screening. As a consequence of these developments, instead of testing for one condition or one gene, tests have become more panel-based [5]. While hereditary disorders are becoming increasingly treatable (e.g., by developments in stem cell transplantation and gene therapy [15]), and/or preventable (e.g., bilateral prophylactic mastectomy in hereditary breast cancer), at the same time innovation in genetic testing is improving opportunities for their early detection. 

In diagnostic settings, DNA sequencing enables a better detection of monogenic conditions whose suspected genetic cause is yet unclear [16]. As costs decrease, whole exome and whole genome sequencing (WES/WGS) is becoming more easily accessible and enables testing for an increasing number of conditions simultaneously. Where in diagnostics these wider gene panels and WES/WGS are becoming increasingly common practice, applications of DNA sequencing in preconception, prenatal and neonatal screening are now also currently being studied or have recently been introduced [6,17,18,19]. In a screening context, with a target population without clinical symptoms, the complex interpretation of genetic variants is particularly challenging and continuously evolving.

In addition to increasing scientific knowledge relevant for the detection of monogenic conditions, research is also being conducted on the use of genetic risk profiles for the better prediction of multi- and polygenic conditions, where wider genetic variant panels may contribute to the risk stratification of current cancer screening programs (e.g., in breast cancer screening) [20]. However, the efficacy, feasibility and acceptability of applying these polygenic risk scores is still largely unknown [21].

New options for screening based on developments in the field of pharmacogenetics are also being suggested. Here, there is increasing evidence that using a pharmacogenetic panel could help prevent adverse drug reactions by informing the dosing and selection of drugs [22]. Pilot studies on pharmacogenetic panels currently focus on high-need populations, e.g., adults receiving drug prescription for one of the relevant therapies [22,23], or newborns admitted to neonatal intensive care units (NICUs) [24]. However, the introduction of pharmacogenetic panels into population-based newborn or prenatal screening is still under debate [25,26]. 

### 2.2. Evidenced-Based Policy Decisions

All offers of screening need to adapt to changing opportunities and demands. High-quality scientific evidence is needed as a basis for policy decision-making [2,4]. Subsequently, innovation in screening will generally require an investment in terms of the organization of education, workforce, facilities and pipelines for evaluation. Where (public) healthcare budgets are tight, this may pose a challenge. Evidence on cost-effectiveness will therefore be even more relevant in order to convince policy-makers to initiate and/or enable change [2]. However, in a reproductive context this may be considered ethically sensitive [27].

### 2.3. Commercialization of Genetic Screening

Parallel to the expansion of screening in public-funded health programs, there has been a rise in direct-to-consumer genetic tests, providing individuals with access to screening tests without involving healthcare professionals [28,29]. This has raised concerns about the need for proper genetic counselling, consent and data privacy [30]. Moreover, the commercialization of genetic testing services raises ethical questions about marketing strategies and profit motives [31].

## 3. Challenges Faced in Screening Tests Offered at Different Phases in Life

**Figure 2:**
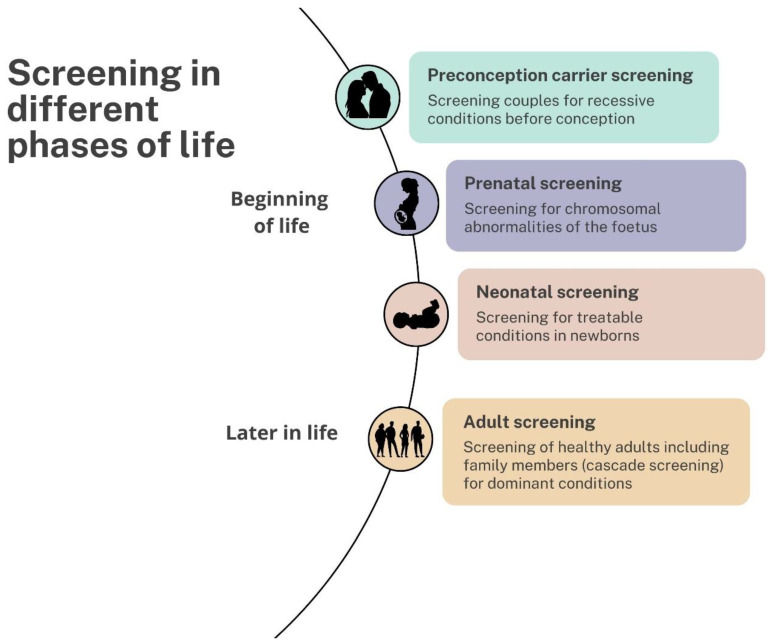
Screening at different phases in life.

Genetic screening programs can be offered at different phases in life (Figure 2). Carrier screening can be offered before pregnancy to assess the risk of couples of having a child affected with recessive genetic conditions. During pregnancy, screening for fetal chromosomal conditions and structural anomalies can be offered to provide pregnant individuals with more information about the health of their unborn child and promote reproductive autonomy. In many countries around the world, newborns undergo screening for treatable conditions to prevent or limit irreparable damage. Later in life, cascade screening in families for monogenic subsets of common disorders enables disease prevention. This involves the diagnosis of individuals with a monogenic disorder (index patient), such as hereditary breast cancer and familial hypercholesterolemia (FH), followed by active identification, counselling and testing of family members. The population screening of adults is currently mainly organized around the early detection of cancer. The use of genetics in these existing adult screening programs is rare, although some suggest that genetic risk-based stratification may improve screening and prevention strategies in this population [20,21]. 

A number of challenges are currently being encountered in the offers of screening at various phases of life. Some of the key challenges will be described here (Table 1).

### 3.1. Challenges in Preconception Carrier Screening: Organizing a Program

Carrier screening for recessive disorders can be defined as the identification of the carrier status of a particular disorder in healthy individuals with no a priori increased risk (i.e., without a positive family history) [32]. It allows couples to know whether they are facing a one-in-four risk of having an affected child in each pregnancy. Carrier screening is preferably performed before pregnancy (preconception) as there is less of a time constraint, and it provides couples with the maximum number of reproductive options, including preimplantation genetic testing. Traditionally, carrier screening has been limited to a small number of relatively common recessive disorders, e.g., β-thalassemia and cystic fibrosis, or was provided to specific high-risk groups with increased risk for disorders based on geographic and/or ethnic background, e.g., in the Ashkenazi Jewish community [33]. Sequencing technologies make carrier screening independent of ancestry ready for implementation by the introduction of expanded carrier screening tests that screen for a large number of recessive disorders simultaneously. In most countries, carrier screening for recessive disorders is not current practice, although commercial providers are offering a wide variety of screening panels, especially in the USA [31], thus limiting screening to those who can afford it. Barriers to the implementation of population-based screening programs have been identified in numerous studies and can be clustered at a cultural, structural and practical level [34]. These barriers include a lack of awareness, knowledge and prioritization among health professionals (e.g., general practitioners) [34,35], a lack of demand from the general population and difficulties reaching the target population (i.e., couples planning a pregnancy, especially in the absence of preconception care facilities) [34,36]. 

Worldwide, there are a wide variety of carrier screening disease panels [31]. It is recommended that these panels should be limited to severe childhood onset conditions, and should not include conditions primarily associated with a disease of adult onset [32,37]. However, in practice, it has proved difficult to define what a “severe disorder” is, as severity can have different dimensions and can be perceived differently depending on the perspective [38]. Practice also shows that the purpose of screening—to increase the reproductive autonomy of couples—is becoming broader, potentially compromising the couples’ informed decision-making. For example, the Australian government-funded pilot project “Mackenzie’s Mission” also included conditions “for which early diagnosis and intervention would substantially change outcome”, meaning that “there is potential benefit from knowing about the condition to inform management in the neonatal period” [19].

Given the large number of carriers identified by expanded screening in the general population, a couple-based approach, wherein only the results of couples who both are carriers of the same disease (1–2% of screened couples) are disclosed, is preferred [33], as was done in Mackenzie’s Mission [19]. This approach not only reduces the psychological burden and the burden on the healthcare system (including genetic counselling costs) [35], but also accelerates variant interpretation [33]. 

### 3.2. Challenges in Prenatal Screening: Ensuring Informed and Autonomous Decision-Making

Prenatal screening offers prospective parents the opportunity to learn information regarding the health of their child, thus enabling them to make informed reproductive choices. The field of prenatal screening has experienced a major paradigm shift over the past decades. The NIPT is a unique example of the “mainstreaming” of a genetic technology. This screening test rapidly passed through the phases of translation, driven by technological developments and the high demand from pregnant women, and was introduced into clinical practice in 2011 [39]. Genetic technologies such as NIPT raise questions about the impact on reproductive choices, governmental regulations and the aim of prenatal screening. 

Initially, NIPT was offered only for the detection of fetal trisomies 21, 18 and 13 (Down, Edwards and Patau syndrome). However, sequencing methods analyzing and reporting findings on all chromosomes soon followed, resulting in an expanding scope. The technological possibilities of NIPT are rapidly expanding, allowing for the screening of other variants such as fetal–maternal risk factors (e.g., preeclampsia) [40] and viral infections (e.g., cytomegalovirus) [41]. However, currently there is no consensus on which other variants should be included in NIPT, and professional societies are warning that caution should be exercised [42,43]. Studies have shown that most pregnant couples would prefer to learn more information about their unborn child [44,45], even to the point of accepting a less accurate test in exchange for receiving this information [46]. Nevertheless, the potential benefits and harms of these expanded tests need to be evaluated, preferably in advance of their introduction into clinical practice [47]. 

An emerging issue within prenatal screening is the blurring of the aims of screening. With expanding offers of testing that also include disorders that are treatable during pregnancy, the aim of prenatal screening is shifting from reproductive autonomy to include health benefit and prevention. Offering dual-aimed screening to pregnant couples in one package may hinder their informed decision-making. NIPT for treatable conditions may also cause a moral shift in societal views on NIPT, from promoting reproductive autonomy to a means to improve the health outcomes for the child [48]. 

Worldwide, NIPT is available to pregnant couples, with varying scopes (ranging from targeted, to varying micro-deletion panels, to genome-wide) and with varying levels of government regulation (ranging from no regulation, fragmented offers of testing, to public screening programs) [49]. The expanding scope of NIPT, rapid developments and lack of government regulation challenge prenatal counselling and provision of information. Offering NIPT within governmentally regulated screening programs is a way to ensure uniform and high-quality information provision and counselling, thus ensuring that parents can make informed and autonomous decisions [50].

### 3.3. Challenges in Neonatal Screening: Maintaining a High-Quality Program

Neonatal bloodspot screening (NBS) programs for newborns are offered shortly after birth to test for an increasing number of treatable disorders, most of which are genetic. Many programs originally started by testing for phenylketonuria, wherein a diet may prevent severe developmental delay. Today, countries screen for a very diverse number of diseases. While neonatal screening is absent in some African countries, many states in the USA screen for over 50 conditions [51]. 

A recent review article stresses the complexity of the NBS infrastructure [8]. In such a public health program, coordination and quality management are very important. Digital data exchange systems have the potential to support NBS programs, but strict data protection regulations may hamper their implementation. For rare conditions included in NBS programs, international databases may serve to achieve sufficient case numbers to evaluate potential new candidate conditions.

An emerging issue in neonatal screening is the question of whether sequencing technologies can replace some, or all, of the current test strategies, since this would also allow for screening for an increasing number of conditions [5]. In case no biomarker is currently available for a disease, sequencing the gene would be the first technological option to allow for screening. King et al. [17] argue that inborn errors of immunity is a field that would profit from sequencing by using a single platform to screen for hundreds of diseases simultaneously. Implementation aspects of newborn sequencing are currently being studied in a variety of pilot projects across the globe, e.g., UK Generation Study and US BabySeq project, as shown on the website www.iconseq.org (accessed on 29 April 2024). Before moving to sequencing as a first-tier test for unselected newborns, its technical and ethical aspects should be reflected upon [2]. Technical questions include how to organize a fast turnaround time, and also the diseases and variants that should be reported on. Should these be pathogenic variants in genes coding for treatable diseases only? How to deal with variants that are associated with late-onset forms of disease? [52] Should variants of uncertain significance (VUS) and carrier status information be avoided? What should happen if the list of pathogenic variants is updated? Could “actionable” conditions such as Fragile X syndrome be added to newborn screening programs [53]? The Fragile X community has expressed a desire for newborn screening. The umbrella organization of rare disease patients EURORDIS [54] has published a similar vision. Parents may value the certainty of prediction over actionability [55]. The expansion of newborn screening to include many more conditions may also result in a decline in overall screening uptake due to the need for more detailed consent [55].

### 3.4. Challenges in Adult Screening: Optimizing Cascade Testing and Deciding on New Opportunities

In recent decades, cascade testing of first-degree family members of patients with mainly autosomal dominant disorders has become standard practice in clinical genetics for early diagnosis and prevention. For some more common disorders, cascade testing for monogenic subsets has attracted substantial attention as well as funding to support more systematic case finding and even screening programs. For example, in Israel, population screening for pathogenic (founder) variants in *BRCA1/2* genes in women of Ashkenazi Jewish origin has been proposed and studied [56]. Especially in the case of *BRCA1/2*-related breast cancer, Lynch syndrome and FH cascade screening has proven to be a cost-effective way to find individuals at high risk to develop these disorders [14]. According to the US Centers for Disease Control and Prevention Office of Genomics and Precision Public Health, such “Tier 1” genomic applications are supported by evidence-based guidelines and recommendations [57]. However, there are gaps in key data [14], and in many countries cascade testing has still not been widely or optimally implemented. Barriers to such implementation may be related to factors at an individual level (e.g., knowledge and attitudes), the interpersonal level (e.g., family dynamics) or a structural level (e.g., accessibility of services) [58]. To optimize implementation and uptake, important challenges that need to be addressed relate to organizational aspects of such screening. The process of case finding and informing family members in a responsible manner has been discussed widely. In recent years, discussions have focused on a more proactive role for healthcare providers to actively trace and inform family members rather than rely on patient-led initiatives to inform family members. In this way, cascade screening uptake is expected to increase; however, national regulations may limit this approach [59]. Digital means of improving the sharing of genetic information in families are also being explored [60].

In some countries, more structured initiatives to improve case finding [61], or actual screening programs to improve finding family members at risk, especially for FH, have been developed [62,63]. Combinations of strategies for case finding and screening have been suggested, e.g., by screening children and subsequently their parents and first-degree family members [64,65]. Another option would be to use the increasing instances of sequencing in healthcare to identify pathogenic variants in a set of well-known genes outside the original indication for screening. Such opportunistic screening may aid in case finding and potentially in subsequent cascade testing. However, much is still unknown about the penetrance of such genes in hitherto unaffected families and the effects of such screening for patients, or about the resources required from healthcare systems to organize such screening [14,66,67]. Identifying if and how such initiatives could be expanded toward forms of population screening requires for many open questions on clinical utility to be further addressed [14,68].

Another recent development is exploring if and how the addition of intermediate or low-risk gene variants to population screening programs may improve health outcomes. In adult-population breast cancer screening, better stratification of both high and low-risk groups could potentially impact the relative burden and/or outcome for participants; therefore, what would be the most cost-effective strategy to apply this knowledge—in what target population and when—is currently under discussion [21,69,70]. This is further complicated by the possibility to optimize prediction by adding non-genetic factors into algorithms, such as breast tissue density in breast cancer screening [69]. 

## 4. Discussion: Challenges and Future Directions in Genetic Screening

We have discussed how dynamics in technology, organization, demand and acceptability change the field of genetic screening at different phases in life. The table clearly shows that some of these dynamics relate to more than one phase in life. Here, we will discuss the commonalities.

### 4.1. Technology

Genetic technologies are becoming faster, cheaper and more precise. An increasing number of tests are becoming available, in addition to new treatments, increasing opportunities for screening, as we have reported previously [5]. Tests have become more genome-based, making it possible to identify more conditions, but also generating more uncertain results. Thus, what to report in screening may become less clear, as the setting is different from that of diagnostics; where in diagnostics patients have a clinical enquiry, screening generally starts from healthy individuals without a pathological phenotype. To maintain a high specificity, there is a need to avoid the reporting of VUS and uncertain genetic variants, as well as variants with low penetrance or unclear onset of disease. In the reproductive context, it is especially important to limit reporting to pathogenic variants [32]. 

Evolving technologies lead to the blurring of aims in screening (reproductive autonomy versus prevention), both in the preconception and prenatal settings. In adult screening, predictability is limited by two factors: the use of lower-risk in addition to high-risk variants and non-genetic risk factors. Ongoing trials will generate evidence for this. As for adult screening, the penetrance of high-risk variants in the absence of family history is not always clear [71]. Stakeholders need to attune and decide together what type of evidence is needed for policy decisions, and how this evidence can best be produced and studied. While some stakeholders may have high expectations of high throughput DNA sequencing replacing existing approaches (e.g., in newborn screening), outcomes of pilots will be needed to feed the discussions on the actual added value [6,72].

### 4.2. Organization

Emerging issues in genetic screening can be identified at the organizational level. Funding for preconception carrier screening may not fit into our healthcare systems, which typically fund the consequences of diseases but not their prevention. Cascade screening for FH may look like a convincing case, but its implementation appears to be problematic. In both preconception and adult screening, it is not clear who is responsible for the implementation of genetic screening, leaving only fragmented ad hoc initiatives. In public health screening programs, there seems to be a gap between public health experts and genetic experts [73].

Neonatal screening programs are complex and continuously evolving systems, requiring good central coordination and digital support systems. The challenges of data storage versus privacy protection become even more intricate when DNA sequencing techniques are proposed in the context of neonatal screening. When large datasets on newborns are being stored, especially when it concerns sensitive data, as is the case with using WGS, re-consent at adolescent/adult age for storage and (secondary) future use will need to be considered and this will involve organizational challenges [74]. The necessary complex data infrastructures for storing large datasets require costly investments and will also have a negative environmental impact. If WGS data are needed again later in life, the declining costs of sequencing will make re-sequencing more feasible, and may therefore be preferred over re-use of existing data.

Commercial testing is already frequently offered in the preconception and prenatal settings. Potentially, this will gain prominence in neonatal and adult screening, and therefore will need good governance of aspects such as quality control and information provision. Unsound initiatives may not always be prevented under current legislations, thus necessitating continuous monitoring of the commercial market and evaluation of legislation [75].

We also show that the benefits and risks of screening often are discussed in relation to cost-effectiveness and distributive justice. Access to preconception and prenatal testing, especially in a private healthcare setting, is limited to people who can afford it. 

In all public health screening programs, equity should be striven for. The development of risk stratification may allow for a better benefit–harm ratio. If a large proportion of potential screenees were to use commercial testing, this may lead to follow-up testing for a selected (i.e., high-income) population, overdiagnosis and overtreatment, which is likely to distort the benefit–harm ratio. 

In addition, distributive justice is even more at stake in low- and middle-income countries. In a country like India, for instance, with its high prevalence of thalassemia patients who are consuming an increasing proportion of the health budget, preconception carrier screening is considered more urgent than ever [76].

### 4.3. Demand

We describe a discrepancy between prenatal and neonatal screening versus preconception and adult screening, whereby the demand in the first domains is increasing and less straightforward for the latter domains. In general, with the increasing focus on the empowerment of citizens and self-management in health and disease, a rising demand for information is to be expected in the future. The limited involvement in preventive genomics of both citizens and professionals, as well as limited genetic literacy, both pose challenges to such empowerment [77,78,79]. 

### 4.4. Acceptability

Many challenges that need to be discussed among stakeholders for policy-making have emerged. Ethical issues relate to what is considered severe and to blurring aims of screening. Non-severe conditions, uncertain test results, variants with diverse penetrance and expression levels, overdiagnosis and overtreatment must all be avoided in a public health program. 

Policy-makers who decide on what should be implemented also have to take into account cost, availability of the required evidence on clinical validity and clinical utility and the benefit–harm ratio.

## 5. Conclusions

The increasing number of technical possibilities with regard to screening tests and treatment possibilities are creating pressure on decision-makers who have to decide on the introduction or expansion of screening. All stakeholders need to become attuned to their priorities in terms of severe diseases and actions after positive test results. A broader implementation of sequencing technologies in screening requires more information in order to make informed choices. We are witnessing a diversification of applications both within healthcare and public health, as well as in commercial settings. In screening, a clear focus on balancing benefits and harms needs to be upheld. Emerging evidence for such benefits and harms should be assessed on the basis of ongoing research and pilot studies, allowing for the careful evaluation and refining of policies. In public health, such balancing requires a focus on impact at the population level, including elements of personal choice and preferences, where possible. Empowering patients to make optimal choices in genetic screening requires a resilient screening infrastructure that also includes strategies to improve genetic health literacy among the wider population. 

## Figures and Tables

**Table 1:** Key challenges of innovation in genetic applications in screening related to sources of dynamics and at different phases of life.

Sources of Dynamics	Screening According to Phases of Life			
	Preconception	Prenatal	Neonatal	Later in Life
Technology	No consensus on which gene variants to include, analyze and report.	Increasing possibilities for screening for other genetic variants (e.g., viruses, feto–maternal risk factors). No consensus on what to include.	Not clear when and where to apply DNA sequencing. Potential increase in treatability of rare genetic diseases (with rapid development of gene therapies).	Limited or unclear predictability of lower risk variants. Unclear penetrance in absence of positive family history.
Organization	Unclear where and how to implement carrier screening. Lack of funding and organizational structures, with high costs of tests.	Rapidly changing field challenges up-to-date counselling and information provision. Fragmentation in test offer and commercialization: need for governmental regulations.	Rapid technological developments need fast and flexible organization and evaluation models.	Unclear where to implement, how and for whom (which subgroups).
Demand	Lack of demand.	Increasing demand for expanding scope.	Continuous demand for expanding the scope to actionable disorders.	Limited demand specifically for genetic testing.
Acceptability	No consensus on what is considered a severe disease. No consensus on the purpose of screening.	Blurring of aims of screening. Expanding scope may challenge acceptability.	Implementing DNA sequencing and expanding scope to actionable conditions may challenge current uptake.	Initial applications as opportunistic offer, challenging equal access and resources needed for follow-up. Clinical utility not yet proven, e.g., of adding low-risk variants in population-based screening offers.

## Data Availability

Not applicable since no new data were created.

## References

[1] Godard B., ten Kate L., Evers-Kiebooms G., Aymé S. (2003). Population genetic screening programmes: Principles, techniques, practices, and policies. Eur. J. Hum. Genet..

[2] Turnbull C., Firth H.V., Wilkie A.O.M., Newman W., Raymond F.L., Tomlinson I., Lachmann R., Wright C.F., Wordsworth S., George A. (2024). Population screening requires robust evidence-genomics is no exception. Lancet.

[3] Wilson J.M.G., Jungner G. (1968). Principles and Practice of Screening for Disease Geneva.

[4] Andermann A., Blancquaert I., Beauchamp S., Déry V. (2008). Revisiting Wilson and Jungner in the genomic age: A review of screening criteria over the past 40 years. Bull. World Health Organ..

[5] Cornel M.C., Rigter T., Jansen M.E., Henneman L. (2021). Neonatal and carrier screening for rare diseases: How innovation challenges screening criteria worldwide. J. Community Genet..

[6] Burki T. (2022). UK explores whole-genome sequencing for newborn babies. Lancet.

[7] Hong D., Zheng Y.-Y., Xin Y., Sun L., Yang H., Lin M.-Y., Liu C., Li B.-N., Zhang Z.-W., Zhuang J. (2021). Genetic syndromes screening by facial recognition technology: VGG-16 screening model construction and evaluation. Orphanet J. Rare Dis..

[8] Odenwald B., Brockow I., Hanauer M., Lüders A., Nennstiel U. (2023). Is Our Newborn Screening Working Well? A Literature Review of Quality Requirements for Newborn Blood Spot Screening (NBS) Infrastructure and Procedures. Int. J. Neonatal Screen..

[9] Milko L.V., Khoury M.J. (2022). Editorial: DNA-based population screening for precision public health. Front. Genet..

[10] Shen E.C., Srinivasan S., Passero L.E., Allen C.G., Dixon M., Foss K., Halliburton B., Milko L.V., Smit A.K., Carlson R. (2022). Barriers and Facilitators for Population Genetic Screening in Healthy Populations: A Systematic Review. Front. Genet..

[11] van Dijke I., van El C.G., Lakeman P., Goddijn M., Rigter T., Cornel M.C., Henneman L. (2022). Dynamics of reproductive genetic technologies: Perspectives of professional stakeholders. PLoS ONE.

[12] van Schendel R.V., van El C.G., Pajkrt E., Henneman L., Cornel M.C. (2017). Implementing non-invasive prenatal testing for aneuploidy in a national healthcare system: Global challenges and national solutions. BMC Health Serv. Res..

[13] Delatycki M.B., Alkuraya F., Archibald A., Castellani C., Cornel M., Grody W.W., Henneman L., Ioannides A.S., Kirk E., Laing N. (2020). International perspectives on the implementation of reproductive carrier screening. Prenat. Diagn..

[14] Murray M.F., Giovanni M.A., Doyle D.L., Harrison S.M., Lyon E., Manickam K., Monaghan K.G., Rasmussen S.A., Scheuner M.T., Palomaki G.E. (2021). DNA-based screening and population health: A points to consider statement for programs and sponsoring organizations from the American College of Medical Genetics and Genomics (ACMG). Genet. Med..

[15] Cavazzana-Calvo M., Hacein-Bey S., de Saint Basile G., Gross F., Yvon E., Nusbaum P., Selz F., Hue C., Certain S., Casanova J.L. (2000). Gene therapy of human severe combined immunodeficiency (SCID)-X1 disease. Science.

[16] Gilissen C., Hoischen A., Brunner H.G., Veltman J.A. (2012). Disease gene identification strategies for exome sequencing. Eur. J. Hum. Genet..

[17] King J.R., Grill K., Hammarström L. (2023). Genomic-Based Newborn Screening for Inborn Errors of Immunity: Practical and Ethical Considerations. Int. J. Neonatal Screen..

[18] van der Meij K.R.M., Sistermans E.A., Macville M.V.E., Stevens S.J.C., Bax C.J., Bekker M.N., Bilardo C.M., Boon E.M.J., Boter M., Diderich K.E.M. (2019). TRIDENT-2: National Implementation of Genome-wide Non-invasive Prenatal Testing as a First-Tier Screening Test in the Netherlands. Am. J. Hum. Genet..

[19] Kirk E.P., Ong R., Boggs K., Hardy T., Righetti S., Kamien B., Roscioli T., Amor D.J., Bakshi M., Chung C.W.T. (2021). Gene selection for the Australian Reproductive Genetic Carrier Screening Project (“Mackenzie’s Mission”). Eur. J. Hum. Genet..

[20] Mavaddat N., Michailidou K., Dennis J., Lush M., Fachal L., Lee A., Tyrer J.P., Chen T.H., Wang Q., Bolla M.K. (2019). Polygenic Risk Scores for Prediction of Breast Cancer and Breast Cancer Subtypes. Am. J. Hum. Genet..

[21] Shieh Y., Eklund M., Madlensky L., Sawyer S.D., Thompson C.K., Stover Fiscalini A., Ziv E., Van’t Veer L.J., Esserman L.J., Tice J.A. (2017). Breast Cancer Screening in the Precision Medicine Era: Risk-Based Screening in a Population-Based Trial. J. Natl. Cancer Inst..

[22] Swen J.J., van der Wouden C.H., Manson L.E., Abdullah-Koolmees H., Blagec K., Blagus T., Böhringer S., Cambon-Thomsen A., Cecchin E., Cheung K.C. (2023). A 12-gene pharmacogenetic panel to prevent adverse drug reactions: An open-label, multicentre, controlled, cluster-randomised crossover implementation study. Lancet.

[23] Jarvis J.P., Peter A.P., Keogh M., Baldasare V., Beanland G.M., Wilkerson Z.T., Kradel S., Shaman J.A. (2022). Real-World Impact of a Pharmacogenomics-Enriched Comprehensive Medication Management Program. J. Pers. Med..

[24] Gallaway K.A., Cann K., Oetting K., Rothenberger M., Raibulet A., Slaven J.E., Suhrie K., Tillman E.M. (2023). The Potential Impact of Preemptive Pharmacogenetic Genotyping in the Neonatal Intensive Care Unit. J. Pediatr..

[25] Ceyhan-Birsoy O., Murry J.B., Machini K., Lebo M.S., Yu T.W., Fayer S., Genetti C.A., Schwartz T.S., Agrawal P.B., Parad R.B. (2019). Interpretation of Genomic Sequencing Results in Healthy and Ill Newborns: Results from the BabySeq Project. Am. J. Hum. Genet..

[26] Raymond M., Critchlow E., Rice S.M., Wodoslawsky S., Berger S.I., Hegde M., Empey P.E., Al-Kouatly H.B. (2022). Fetal pharmacogenomics: A promising addition to complex neonatal care. Mol. Genet. Metab..

[27] Schmidtke J., Cornel M.C. (2020). Contentious ethical issues in community genetics: Let’s talk about them. J. Community Genet..

[28] Horton R., Crawford G., Freeman L., Fenwick A., Wright C.F., Lucassen A. (2019). Direct-to-consumer genetic testing. BMJ.

[29] Su P. (2013). Direct-to-consumer genetic testing: A comprehensive view. Yale J. Biol. Med..

[30] Majumder M.A., Guerrini C.J., McGuire A.L. (2021). Direct-to-Consumer Genetic Testing: Value and Risk. Annu. Rev. Med..

[31] Chokoshvili D., Borry P., Vears D.F. (2018). A systematic analysis of online marketing materials used by providers of expanded carrier screening. Genet. Med..

[32] Henneman L., Borry P., Chokoshvili D., Cornel M.C., van El C.G., Forzano F., Hall A., Howard H.C., Janssens S., Kayserili H. (2016). Responsible implementation of expanded carrier screening. Eur. J. Hum. Genet..

[33] Antonarakis S.E. (2019). Carrier screening for recessive disorders. Nat. Rev. Genet..

[34] Holtkamp K.C., Vos E.M., Rigter T., Lakeman P., Henneman L., Cornel M.C. (2017). Stakeholder perspectives on the implementation of genetic carrier screening in a changing landscape. BMC Health Serv. Res..

[35] Fehlberg Z., Best S., Long J.C., Theodorou T., Pope C., Hibbert P., Williams S., Freeman L., Righetti S., Archibald A.D. (2023). Scaling-up and future sustainability of a national reproductive genetic carrier screening program. NPJ Genom. Med..

[36] Best S., Long J., Theodorou T., Hatem S., Lake R., Archibald A., Freeman L., Braithwaite J. (2021). Health practitioners’ perceptions of the barriers and enablers to the implementation of reproductive genetic carrier screening: A systematic review. Prenat. Diagn..

[37] Gregg A.R., Aarabi M., Klugman S., Leach N.T., Bashford M.T., Goldwaser T., Chen E., Sparks T.N., Reddi H.V., Rajkovic A. (2021). Screening for autosomal recessive and X-linked conditions during pregnancy and preconception: A practice resource of the American College of Medical Genetics and Genomics (ACMG). Genet. Med..

[38] Dive L., Archibald A.D., Freeman L., Newson A.J. (2023). How should severity be understood in the context of reproductive genetic carrier screening?. Bioethics.

[39] Dondorp W., de Wert G., Bombard Y., Bianchi D.W., Bergmann C., Borry P., Chitty L.S., Fellmann F., Forzano F., Hall A. (2015). Non-invasive prenatal testing for aneuploidy and beyond: Challenges of responsible innovation in prenatal screening. Eur. J. Hum. Genet..

[40] Rasmussen M., Reddy M., Nolan R., Camunas-Soler J., Khodursky A., Scheller N.M., Cantonwine D.E., Engelbrechtsen L., Mi J.D., Dutta A. (2022). RNA profiles reveal signatures of future health and disease in pregnancy. Nature.

[41] Faas B.H.W., Astuti G., Melchers W.J.G., Reuss A., Gilissen C., Macville M.V.E., Ghesquiere S.A.I., Houben L.M.H., Srebniak M.I., Geeven G. (2024). Early detection of active Human CytomegaloVirus (hCMV) infection in pregnant women using data generated for noninvasive fetal aneuploidy testing. eBioMedicine.

[42] Hui L., Ellis K., Mayen D., Pertile M.D., Reimers R., Sun L., Vermeesch J., Vora N.L., Chitty L.S. (2023). Position statement from the International Society for Prenatal Diagnosis on the use of non-invasive prenatal testing for the detection of fetal chromosomal conditions in singleton pregnancies. Prenat. Diagn..

[43] Dungan J.S., Klugman S., Darilek S., Malinowski J., Akkari Y.M.N., Monaghan K.G., Erwin A., Best R.G. (2023). Noninvasive prenatal screening (NIPS) for fetal chromosome abnormalities in a general-risk population: An evidence-based clinical guideline of the American College of Medical Genetics and Genomics (ACMG). Genet. Med..

[44] van der Meij K.R.M., van de Pol Q.Y.F., Bekker M.N., Martin L., Gitsels-van der Wal J., van Vliet-Lachotzki E.H., Weiss J.M., Galjaard R.H., Sistermans E.A., Macville M.V.E. (2023). Experiences of pregnant women with genome-wide non-invasive prenatal testing in a national screening program. Eur. J. Hum. Genet..

[45] Dubois M.L., Winters P.D., Rodrigue M.A., Gekas J. (2023). Patient attitudes and preferences about expanded noninvasive prenatal testing. Front. Genet..

[46] Hill M., Johnson J.A., Langlois S., Lee H., Winsor S., Dineley B., Horniachek M., Lalatta F., Ronzoni L., Barrett A.N. (2016). Preferences for prenatal tests for Down syndrome: An international comparison of the views of pregnant women and health professionals. Eur. J. Hum. Genet..

[47] Baldus M. (2023). “Overestimated technology—Underestimated consequences”—Reflections on risks, ethical conflicts, and social disparities in the handling of non-invasive prenatal tests (NIPTs). Med. Health Care Philos..

[48] Garcia E., Henneman L., Gitsels-van der Wal J.T., Martin L., Koopmanschap I., Bekker M.N., Timmermans D.R.M. (2022). Non-invasive prenatal testing (NIPT) and pregnant women’s views on good motherhood: A qualitative study. Eur. J. Hum. Genet..

[49] Ravitsky V., Roy M.C., Haidar H., Henneman L., Marshall J., Newson A.J., Ngan O.M.Y., Nov-Klaiman T. (2021). The Emergence and Global Spread of Noninvasive Prenatal Testing. Annu. Rev. Genom. Hum. Genet..

[50] Lannoo L., van der Meij K.R.M., Bekker M.N., De Catte L., Deckers S., Devriendt K., Roggen N., Galjaard R.H., Gitsels-van der Wal J., Macville M.V.E. (2023). A cross-country comparison of pregnant women’s decision-making and perspectives when opting for non-invasive prenatal testing in the Netherlands and Belgium. Prenat. Diagn..

[51] Therrell B.L., Padilla C.D., Loeber J.G., Kneisser I., Saadallah A., Borrajo G.J., Adams J. (2015). Current status of newborn screening worldwide: 2015. Semin. Perinatol..

[52] Goldstein J.L., McGlaughon J., Kanavy D., Goomber S., Pan Y., Deml B., Donti T., Kearns L., Seifert B.A., Schachter M. (2023). Variant Classification for Pompe disease; ACMG/AMP specifications from the ClinGen Lysosomal Diseases Variant Curation Expert Panel. Mol. Genet. Metab..

[53] Johnson K., Stanfield A.C., Scerif G., McKechanie A., Clarke A., Herring J., Smith K., Crawford H. (2024). A holistic approach to fragile X syndrome integrated guidance for person-centred care. J. Appl. Res. Intellect. Disabil..

[54] EURORDIS Key Principles for Newborn Screening. https://www.eurordis.org/publications/key-principles-for-newborn-screening/.

[55] Downie L., Halliday J., Lewis S., Amor D.J. (2021). Principles of Genomic Newborn Screening Programs: A Systematic Review. JAMA Netw. Open.

[56] Greenberg R., Aharonov-Majar E., Isakov O., Hayek S., Elefant N., Balicer R.D., Berliner Senderey A., Ben-Shachar S. (2023). Carrier screening program for *BRCA1*/*BRCA2* pathogenic variants among Ashkenazi Jewish women in Israel: An observational study. Genet. Med. Open.

[57] Khoury M.J., Feero W.G., Chambers D.A., Brody L.C., Aziz N., Green R.C., Janssens A., Murray M.F., Rodriguez L.L., Rutter J.L. (2018). A collaborative translational research framework for evaluating and implementing the appropriate use of human genome sequencing to improve health. PLoS Med..

[58] Srinivasan S., Won N.Y., Dotson W.D., Wright S.T., Roberts M.C. (2020). Barriers and facilitators for cascade testing in genetic conditions: A systematic review. Eur. J. Hum. Genet..

[59] Roberts M.C., Dotson W.D., DeVore C.S., Bednar E.M., Bowen D.J., Ganiats T.G., Green R.F., Hurst G.M., Philp A.R., Ricker C.N. (2018). Delivery Of Cascade Screening For Hereditary Conditions: A Scoping Review Of The Literature. Health Aff..

[60] Lee W., Shickh S., Assamad D., Luca S., Clausen M., Somerville C., Tafler A., Shaw A., Hayeems R., Bombard Y. (2023). Patient-facing digital tools for delivering genetic services: A systematic review. J. Med. Genet..

[61] Silva L., Qureshi N., Abdul-Hamid H., Weng S., Kai J., Leonardi-Bee J. (2021). Systematic Identification of Familial Hypercholesterolaemia in Primary Care-A Systematic Review. J. Pers. Med..

[62] Leren T.P., Bogsrud M.P. (2021). The importance of cascade genetic screening for diagnosing autosomal dominant hypercholesterolemia: Results from twenty years of a national screening program in Norway. J. Clin. Lipidol..

[63] Louter L., Defesche J., Roeters van Lennep J. (2017). Cascade screening for familial hypercholesterolemia: Practical consequences. Atheroscler. Suppl..

[64] Wald D.S., Bestwick J.P. (2020). Reaching detection targets in familial hypercholesterolaemia: Comparison of identification strategies. Atherosclerosis.

[65] Watts G.F., Gidding S.S., Hegele R.A., Raal F.J., Sturm A.C., Jones L.K., Sarkies M.N., Al-Rasadi K., Blom D.J., Daccord M. (2023). International Atherosclerosis Society guidance for implementing best practice in the care of familial hypercholesterolaemia. Nat. Rev. Cardiol..

[66] de Wert G., Dondorp W., Clarke A., Dequeker E.M.C., Cordier C., Deans Z., van El C.G., Fellmann F., Hastings R., Hentze S. (2021). Opportunistic genomic screening. Recommendations of the European Society of Human Genetics. Eur. J. Hum. Genet..

[67] Ibrahim S., Reeskamp L.F., Stroes E.S.G., Watts G.F. (2020). Advances, gaps and opportunities in the detection of familial hypercholesterolemia: Overview of current and future screening and detection methods. Curr. Opin. Lipidol..

[68] Khoury M.J., Dotson W.D. (2021). From genes to public health: Are we ready for DNA-based population screening?. Genet. Med..

[69] Evans D.G.R., van Veen E.M., Harkness E.F., Brentnall A.R., Astley S.M., Byers H., Woodward E.R., Sampson S., Southworth J., Howell S.J. (2022). Breast cancer risk stratification in women of screening age: Incremental effects of adding mammographic density, polygenic risk, and a gene panel. Genet. Med..

[70] Hingorani A.D., Gratton J., Finan C., Schmidt A.F., Patel R., Sofat R., Kuan V., Langenberg C., Hemingway H., Morris J.K. (2023). Performance of polygenic risk scores in screening, prediction, and risk stratification: Secondary analysis of data in the Polygenic Score Catalog. BMJ Med..

[71] van Rooij J., Arp P., Broer L., Verlouw J., van Rooij F., Kraaij R., Uitterlinden A., Verkerk A. (2020). Reduced penetrance of pathogenic ACMG variants in a deeply phenotyped cohort study and evaluation of ClinVar classification over time. Genet. Med..

[72] Rahimzadeh V., Friedman J.M., de Wert G., Knoppers B.M. (2022). Exome/Genome-Wide Testing in Newborn Screening: A Proportionate Path Forward. Front. Genet..

[73] Rosso A., Pitini E., D’Andrea E., Di Marco M., Unim B., Baccolini V., De Vito C., Marzuillo C., Barnhoorn F., Zeegers Paget D. (2020). Genomics knowledge and attitudes among European public health professionals: Results of a cross-sectional survey. PLoS ONE.

[74] Peay H.L., Gwaltney A.Y., Moultrie R., Cope H., Boyea B.L., Porter K.A., Duparc M., Alexander A.A., Biesecker B.B., Isiaq A. (2022). Education and Consent for Population-Based DNA Screening: A Mixed-Methods Evaluation of the Early Check Newborn Screening Pilot Study. Front. Genet..

[75] Cernat A., Bashir N.S., Ungar W.J. (2022). Considerations for developing regulations for direct-to-consumer genetic testing: A scoping review using the 3-I framework. J. Community Genet..

[76] Sinha S., Seth T., Colah R.B., Bittles A.H. (2019). Haemoglobinopathies in India: Estimates of blood requirements and treatment costs for the decade 2017–2026. J Community Genet.

[77] van Kessel R., Wong B.L.H., Clemens T., Brand H. (2022). Digital health literacy as a super determinant of health: More than simply the sum of its parts. Internet Interv..

[78] Murtagh M.J., Machirori M., Gaff C.L., Blell M.T., de Vries J., Doerr M., Dove E.S., Duncanson A., Hastings Ward J., Hendricks-Sturrup R. (2021). Engaged genomic science produces better and fairer outcomes: An engagement framework for engaging and involving participants, patients and publics in genomics research and healthcare implementation. Wellcome Open Res..

[79] Likhanov M., Zakharov I., Awofala A., Ogundele O., Selita F., Kovas Y., Chapman R. (2023). Attitudes towards genetic testing: The role of genetic literacy, motivated cognition, and socio-demographic characteristics. PLoS ONE.

